# Supplementary material for: CanCOG®: Cultural Adaptation of the Evidence-Based UCLA Cognitive Rehabilitation Intervention Program for Cancer Survivors in Portugal
Source: Healthcare (Basel). 2023 Jan 2;11(1):141. doi: 10.3390/healthcare11010141 (PMC9819200; doi:10.3390/healthcare11010141)
Supplement: Supplementary file 1 [file healthcare-11-00141-s001.zip › Supplementary Material 1.pdf]

Table S1. Interview script.

	Questions
**1**	Do you think this program will be good and useful for you? Do you think it could be beneficial for your daily life in general and work in particular?
**2**	Would you like to participate in a program like CanCOG? In your opinion, what could motivate you to participate in the program?
**3**	What could be a barrier to your participation in CanCOG? What could make you withdraw?
**4**	What would you like to know about cognitive difficulties related to cancer and cancer treatments? What tips/information would be important?
**5**	What is your opinion about the program materials?
**6**	What is your opinion about the program's cognitive exercises? Do you find them difficult? Are there any exercises that are easier/difficult? Would you add any exercise/activity to the program?
**7**	Would you feel comfortable participating in this program in a face-to-face group format, as it was developed?
**8**	Would you participate in this program if it was delivered online? That is, individually, using an online platform, being able to chat with other cancer survivors who are participating at the same time.
**9**	Considering the two formats, which would you prefer?
**10**	What is your opinion on the frequency and duration of the sessions (1 session per week, for 5 weeks, for 2 hours)?
**11**	Regarding homework exercises, as a complement to the exercises performed during the sessions, at least 20 minutes 4x/week are recommended. What is your opinion on this recommendation? Do you consider it feasible given your daily routines?
