# Supplementary material for: CanCOG®: Cultural Adaptation of the Evidence-Based UCLA Cognitive Rehabilitation Intervention Program for Cancer Survivors in Portugal
Source: Healthcare (Basel). 2023 Jan 2;11(1):141. doi: 10.3390/healthcare11010141 (PMC9819200; doi:10.3390/healthcare11010141)
Supplement: Supplementary file 1 [file healthcare-11-00141-s001.zip › Supplementary Material 2.pdf]

Table S2. COREQ (COnsolidated criteria for REporting Qualitative research) Checklist.

Topic	Item No.	Guide Questions/ Description	Reported on Page No.	Explanation
**Domain 1: Research team and reflexivity**				
**Personal characteristics**				
Interviewer/facilitator	1	Which author/s conducted the interview or focus group?	12	A.F.O. and A.B. conducted the interviews.
Credentials	2	What were the researcher's credentials? E.g., PhD, MD	NR	A.F.O. MSc in Health Psychology and Neuropsychological Rehabilitation.<br>A.B. PhD in Psychology.
Occupation	3	What was their occupation at the time of the study?	NR	A.F.O. employed as a PhD candidate.<br>A.B. employed as an assistant professor.
Gender	4	Was the researcher male or female?	NR	A.F.O. and A.B. females.
Experience and training	5	What experience or training did the researcher have?	12	Focus groups were conducted by psychologists with experience in qualitative research.
**Relationship with participants**				
Relationship established	6	Was a relationship established prior to study commencement?	11	Some participants had previous contact with the interviewers before starting the study.
Participant knowledge of the interviewer	7	What did the participants know about the researcher? e.g., personal goals, reasons for doing the research?	NR	Participants knew that the leading researcher was a psychologist undertaking a PhD program.
Interviewer characteristics	8	What characteristics were reported about the interviewer/ facilitator? e.g., Bias, assumptions, reasons and interests in the research topic	NR	The interviewers had experience working and researching with cancer survivors, but none had personally experienced a cancer diagnosis.

**Domain 2: Study design**				
**Theoretical framework**				
Methodological orientation and theory	9	What methodological orientation was stated to underpin the study? e.g., grounded theory, discourse analysis, ethnography, phenomenology, content analysis	10, 12	A qualitative design was followed; namely, a focus group with thematic analysis to explore the program's acceptability.
**Participant selection**				
Sampling	10	How were participants selected? e.g., purposive, convenience, consecutive, snowball	11	Recruitment was carried out using a snowball procedure.
Method of approach	11	How were participants approached? e.g., face-to-face, telephone, mail, email	11	Participants were approached by e-mail.
Sample size	12	How many participants were in the study?	10	Eleven cancer survivors participated in this study.
Non-participation	13	How many people refused to participate or dropped out? Reasons?	10	All contacted cancer survivors voluntarily agreed to participate.
**Setting**				
Setting of data collection	14	Where was the data collected? e.g., home, clinic, workplace	10	The focus groups were conducted online via Zoom.
Presence of nonparticipants	15	Was anyone else present besides the participants and researchers?	10	Only participants and researchers were present.
Description of sample	16	What are the important characteristics of the sample? e.g., demographic data, date	21-22	Details of the sample are included in section "3.3. Appraisal by the Target Population".

**Data collection**				
Interview guide	17	Were questions, prompts, guides provided by the authors? Was it pilot tested?	11-12 Table S1	The focus group agenda and semi-structured guide are reported in the text.
Repeat interviews	18	Were repeat interviews carried out? If yes, how many?	No	Repeat interviews were not conducted.
Audio/visual recording	19	Did the research use audio or visual recording to collect the data?	12	Focus group interviews were visual and audio-recorded.
Field notes	20	Were field notes made during and/or after the interview or focus group?	12	After the focus groups.
Duration	21	What was the duration of the interviews or focus group?	10	The focus groups lasted, on average, 120 minutes.
Data saturation	22	Was data saturation discussed?	No	Data saturation was not discussed.
Transcripts returned	23	Were transcripts returned to participants for comment and/or correction?	No	Transcripts were not returned to participants.
**Domain 3: Analysis and findings**				
**Data analysis**				
Number of data coders	24	How many data coders coded the data?	12	Thematic analysis was carried out independently by two of the researchers (A.F.O. and A.B.).
Description of the coding tree	25	Did authors provide a description of the coding tree?	No	No description of the coding tree is provided.
Derivation of themes	26	Were themes identified in advance or derived from the data?	11-12 Table S1	The focus group guide script provided a framework for data analysis.
Software	27	What software, if applicable, was used to manage the data?	12	The software ATLAS.ti 22 was used.
Participant checking	28	Did participants provide feedback on the findings?	No	Participants were not requested to give feedback on the findings.

Reporting				
Quotations presented	29	-Were participant quotations presented to illustrate the themes/findings?<br>-Was each quotation identified? e.g., participant number	21-22	Quotations are presented to illustrate the findings, along with the participants' sex and age, to preserve anonymity.
Data and findings consistent	30	Was there consistency between the data presented and the findings?	21-22	We aimed to clarify the data presented and the findings, supporting them with direct quotations from the participants.
Clarity of major themes	31	Were major themes clearly presented in the findings?	21-22	Themes are clearly presented in the text.
Clarity of minor themes	32	Is there a description of diverse cases or discussion of minor themes?	No	There is no description of diverse cases or minor themes.
