# Supplementary material for: CanCOG®: Cultural Adaptation of the Evidence-Based UCLA Cognitive Rehabilitation Intervention Program for Cancer Survivors in Portugal
Source: Healthcare (Basel). 2023 Jan 2;11(1):141. doi: 10.3390/healthcare11010141 (PMC9819200; doi:10.3390/healthcare11010141)
Supplement: Supplementary file 1 [file healthcare-11-00141-s001.zip › Supplementary Material 3.pdf]

Table S3. Group characteristics of the UCLA program participants (US) and potential participants from Portugal.

	UCLA program participants – US	Cultural adaptation group – Portugal	Potential mismatch
**Characteristics**			
**Target population**	Female breast cancer survivors aged 21 to 75 years	Adult non-CNS cancer survivors	Type of cancer and treatment used
**Primary language**	English	Portuguese	Language differences may impact understanding and access to intervention
**Country of origin**	US	Portugal	Differences in life experiences, social norms, attitudes, beliefs, and cultural factors
**Setting**	Los Angeles (US) – Urban	Aveiro/Coimbra/Porto/other cities (Portugal) – Urban	n/a
**Sociodemographic characteristics of the populations**			
**Mean Age (years)**	53.8	60.4 *	Sociodemographic differences, mainly related to age and education level, can influence the implementation and validation of the culturally adapted program
**Education level**	Less than college: 25%<br>College graduate: 21%<br>Post-college degree: 54%	Less than college: 68% *<br>College or post-college degree: 32% *	
**Staff**			
**Type of staff**	Trained staff, English-speaking	Trained staff, Portuguese-speaking	n/a
**Community engagement**			
**Community consultation**	Consultation with experts in program design and delivery	Consultation with experts and the target population during the cultural adaptation process	n/a

* Based on Torres, A., Pereira, A., & Monteiro, S. (2012). Cognitive-Behavioral Group Therapy for Breast Cancer Survivors: Description of a Program and Preliminary Evaluation of its Effectiveness. *Psychology, Community & Health, 1*(3), 300–312. https://doi.org/10.5964/pch.v1i3.55
